# Management of Whiplash Associated Disorders in Australian general practice

**DOI:** 10.1186/s12891-017-1899-0

**Published:** 2017-12-29

**Authors:** Jane Nikles, Michael Yelland, Clare Bayram, Graeme Miller, Michele Sterling

**Affiliations:** 1 RECOVER Injury Research Centre NHMRC Centre of Research Excellence in Recovery Following Road Traffic Injuries The University of Queensland, Herston, Australia; 2 School of Medicine, Griffith University, Brisbane, Qld Australia; 3 Family Medicine Research Centre, Sydney School of Public Health, University of Sydney, Sydney, Australia

**Keywords:** General practitioner, Management, Road traffic crash, Injury, BEACH study, Neck pain, Whiplash associated disorders

## Abstract

**Background:**

Whiplash Associated Disorders (WAD) are common and costly, and are usually managed initially by general practitioners (GPs). How GPs manage WAD is largely unstudied, though there are clinical guidelines. Our aim was to ascertain the rate of management (percentage of encounters) of WAD among patients attending Australian general practice, and to review management of these problems, including imaging, medications and other treatments.

**Methods:**

We analysed data from 2013 to 2016 collected by different random samples of approximately 1000 general practitioners (GPs) per year. Each GP collected data about 100 consecutive consultations for BEACH (Bettering the Evaluation and Care of Health), an Australian national study of general practice encounters.

Main outcome measures were: the proportion of encounters involving management of WAD; management including imaging, medications and other treatments given; appropriateness of treatment assessed against published clinical guidelines.

**Results:**

Of 291,100 encounters from 2919 GP participants (a nationally representative sample), WAD were managed at 137 encounters by 124 GPs (0.047%). Management rates were 0.050% (females) and 0.043% (males). For 63 new cases (46%), 19 imaging tests were ordered, most commonly neck/cervical spine x-ray (52.6% of tests for new cases), and neck/cervical spine CT scan (31.6%). One or more medications were prescribed/supplied for 53.3% of WAD. NSAIDs (11.7 per 100 WAD problems) and compound analgesics containing paracetamol and opioids (10.2 per 100 WAD problems) were the commonest medications used by GPs overall. Paracetamol alone was used in 8 per 100 WAD problems. The most frequent clinical/procedural treatments for WAD were physical medicine/rehabilitation (16.1 per 100 WAD problems), counselling (6.6), and general advice/education (5.8).

**Conclusions:**

GPs refer about 30% of new cases for imaging (possibly overutilising imaging), and prescribe a range of drugs, approximately 22% of which are outside clinical guidelines. These findings suggest a need for further education of GPs, including indications for imaging after whiplash injury, identification of those more likely to develop chronic WAD, and medication management guidelines. WAD carry a large personal and economic burden, so the impact of improvements in GP management is potentially significant.

## Background

Motor Vehicle Crashes (MVCs) are the cause of 50 million injuries worldwide and nearly four million emergency department (ED) consultations annually in the US [1, 2]. The most frequent injury resulting from MVCs in the majority of Western countries is Whiplash Associated Disorders (WAD) [3]. During the last 30 years, the cumulative incidence of WAD resulting from MVCs has risen to >300/100,000 people in North America and Western Europe [4]^.^ In Australia, the annual incidence of whiplash injuries is 106 per 100,000 and they comprise ~75% of all survivable MVC injuries [5] with total costs of over $950 million per annum [6, 7]. In Queensland, costs are greater than the combined costs for spinal cord and traumatic brain injury [5].

After being medically evaluated, approximately 90% of those who present to ED after MVC return home. [8]. Only around 50% of those with WAD will fully recover, with 30% remaining moderately to severely disabled [9], creating significant personal, economic, and social distress. Worldwide, chronic pain following MVC is a significant burden and a frequent and expensive public health problem.

The majority of people with WAD will be at some stage seen in primary care by a general practitioner (GP). Australian State Insurance Regulatory Authority Guidelines (a definitive current clinical guideline) recommend that “*the mainstay of management for acute WAD is the provision of advice, encouraging return to usual activity, and exercise*” [10]. Following general pain management guidelines is recommended when prescribing medication for these patients. This recommendation is consensus based because there is sparse evidence on the pharmacologic treatments for WAD [11]. “*Simple analgesics may be used as first line treatment for pain relief. NSAIDs may be used if simple analgesics are ineffective. Oral opioids may be necessary to relieve severe pain. Ongoing need for such treatment requires reassessment* [10].*”* No available evidence supports the use of paracetamol, nonsteroidal anti-inflammatory drugs (NSAIDs), or opioids in the management of WAD [11]. The latter two drugs, in particular, need to be used cautiously because of the potential for adverse events, particularly in the elderly.

In Australia, as in certain other countries such as The Netherlands, Norway and Sweden, GPs provide the majority of primary care and can refer patients to other medical specialists. Medicare, Australia’s national medical insurance scheme, covers (partly or completely) the cost of doctor visits. Multidisciplinary chronic disease care plans, coordinated by GPs, are encouraged. Since 2005, Medicare will provide partial reimbursement for 5 visits per year to allied health professionals. Most cases of WAD will be managed under a Compulsory Third Party or workers compensation scheme. Medicare will seek the funds back from the insurer. However, in order to submit a claim to these schemes, patients need to see a GP.

A recent Australian cross-sectional survey [12] explored GPs’ knowledge, attitudes and usual practice relating to diagnosis and management of WAD. Overall, GPs’ knowledge about WAD was good; only 9.6% (95% CI: 7.1–12.8) had lower level knowledge. However, a key knowledge gap was indications for imaging. GPs reported commonly referring to physiotherapists and least frequently to vocational rehabilitation providers [12]. This study described GPs’ knowledge and attitudes to management of WAD, but did not describe their actual management of WAD, and did not address GPs’ pharmacological management.

In Australia, GPs see about 66% of those injured in motor vehicle crashes [12]. There is limited detailed information about how GPs manage WAD, either in Australia or overseas. Little research has been done on the use of medication for WAD in Australian general practice, in particular whether it is prescribed/recommended according to published clinical guidelines.

In summary, WAD is common and costly with chronic pain being a common outcome. General practice is usually the first patient contact point in the healthcare system. GPs play an important gate-keeper role but there is a knowledge gap about how GPs manage these patients. Our aim in this study, therefore, was to document the management of WAD in general practice in Australia, and in particular, to ascertain:

- the percentage of Australian general practice consultations involving management of WAD problems;
- treatments being provided/recommended by GPs;
- the medications prescribed for these problems; and
- if management provided is consistent with current clinical practice guidelines.

## Methods

The BEACH (Bettering the Evaluation and Care of Health) program was an Australian national study of general practice encounters from 1998 to 2015 [13]. Each year a new random sample of 1000 GPs, drawn from Medicare claims data by the Australian Government Department of Health, were invited to complete a questionnaire about themselves and their practice, and record diagnosis and management details for each of 100 consecutive consultations on structured paper forms. Management actions recorded included: medications (up to 4 per problem or 16 per encounter), referrals (up to 3 per encounter), clinical and procedural treatments (up to 2 per problem, 8 per encounter), imaging (up to 3 per encounter) and pathology (up to 5 per encounter) ordered. GPs linked each management action directly to the specific problem being managed on the recording form. In 2013–2014 approximately 85.2% of the Australian population claimed at least one GP service from Medicare. The average number of visits per person annually was 7 [13].

### Classification of problem managed

Problems managed and clinical treatments were secondarily classified from the free text GP description. WAD is classified in the International Classification of Primary Care-2 (ICPC-2) [14] as L79 (Sprains and strains of joints [not otherwise classified]). The more specific term ‘injury:neck:whiplash’ is coded as L79 042 in the Australian general practice clinical terminology (known as ICPC-2 Plus [15]).

### Classification of medications

All medications used in the management of WAD were secondarily classified using the Anatomical Therapeutic Chemical (ATC) classification [16]. GPs were permitted to record up to four medications per WAD problem.

### Statistical methods

A cluster sample design was used, with the GP as the primary sampling unit, and the encounter the primary unit of inference. Using procedures in SAS software (version 9.3, SAS Institute, Cary, NC, USA), 95% confidence intervals were calculated, allowing for the cluster sample design.

## Results

Details about 291,100 encounters were recorded by 2919 GPs from April 2013 to March 2016. WAD problems were recorded by 124 GPs for 137 patients (0.047% of encounters). This equates to an estimated 65,000 occasions of WAD management in Australian general practice per year. Sixty-three (43%) were new presentations and 74 (54%) were previously diagnosed (old) cases.

The characteristics of these 137 patients are given in Table 1.

**Table 1:** Characteristics of the 137 patients with WAD managed at encounter

Data	n	% of WAD encounters (95% CI)
Sex *(Missing)*	*(1)*	
Male	50	36.8 (28.2–45.4)
Female	86	63.2 (54.6–71.8)
Age *(Missing)*	*(2)*	
5–14 years	2	1.5 (0–3.6)
15–24 years	24	17.8 (11–24.5)
25–44 years	58	43 (34.2–51.7)
45–64 years	37	27.4 (19.9–35)
65–74 years	11	8.1 (3–13.2)
75+ years	3	2.2 (0–4.8)
Background *(Missing)*	*(12)*	–
Non-English–speaking background	18	14.4 (7.5–21.3)
English-speaking background	107	85.6 (78.7–92.5)
Rurality^a^ *(Missing)*	*(2)*	
Major city	109	80.7 (73.7–87.8)
Inner regional city	19	14.1 (8–20.2)
Outer regional city	7	5.2 (0.9–9.5)
Missing values	2	.
Commonwealth concession cards *(Missing)*	*(10)*	
Card holders	37	29.1 (20.9–37.3)
Non-card holders	90	70.9 (62.7–79.1)

^a^Australian standard geographical classification [17]

WAD were managed at 0.05% of encounters with female patients, and 0.04% of those with males. Age-specific rates were lowest among children aged under 15 years (<0.01%). The highest management rates were in those aged 15–24 years (0.11%, 95% CI 0.06–0.15%), and those aged 25–44 years (0.09%, 95% CI 0.07–0.12%)), compared with the age groups over 45 years (0.05% or less, 95% CI 0.03–0.06).

Patients with Commonwealth concession cards were less likely to have WAD managed (3.1%, 95% CI: 2.0–4.1) than non-card holders (6.2%, 95% CI: 4.8–7.5). Patients living in major cities were more likely to have WAD managed (5.6%, 95% CI: 4.4–6.7) than those living in inner/outer regional areas (2.9%, 95% CI: 1.7–4.1).

### Management of WAD

Table 2 shows that at least one non phamacological (clinical or procedural) treatment was provided for 36.5% of WAD problems managed. One or more imaging tests were ordered for 25 (18.2%), and at least one pathology test for 1 (0.7%). GPs referred 25.5% of WAD cases, most commonly to allied health services (22.6 per 100 WAD).

**Table 2:** GP management of Whiplash Associated Disorders

Management action	n	% of WAD problems (95% CI)
At least 1 medication	73	53.3 (44.4–62.2)
At least 1 other treatment	50	36.5 (28.2–44.7)
At least 1 referral	35	25.5 (17.9–33.2)
At least 1 imaging test	25	18.2 (11.2–25.3)
	n	Rate per 100 WAD problems managed
Medications	103	75.2 (57.7–92.7)
Prescribed	74	54.0 (36.0–72.0)
Advised OTC	27	19.7 (11.7–27.7)
GP supplied	2	1.5^a^
Other treatments	64	46.7 (34.9–58.5)
^b^Clinical	37	27 (18.8–35.2)
^c^Procedural	27	19.7 (11–28.4)
Referrals	35	25.5 (17.9–33.2)
Specialist	2	1.5^a^
Allied health services	31	22.6 (15.0–30.2)
Other referral	2	1.5^a^
Imaging	29	21.2 (12.7–29.6)
Diagnostic radiology	17	12.4 (6.0–18.8)
Computerised tomography	10	7.3 (1.7–12.9)
Magnetic resonance imaging	2	1.5^a^

CI: 95% confidence interval; OTC: advised over-the-counter medication

^a^95% CIs not calculable due to low frequency

^b^Clinical treatments include advice, education, counselling, reassurance

^c^Procedures include all physical treatments (i.e. manual therapy, injection and splinting)

#### Imaging

Of 29 imaging tests ordered (21.2 per 100 WAD cases), the most common were: neck/cervical spine x-ray (48.3%), neck/cervical spine CT scan (27.5%), and lumbar spine CT scan (6.9%).

For 63 new WAD cases, 19 imaging tests were ordered (30.2 per 100 new WAD cases), most commonly neck/cervical spine x-ray (52.6% of tests for new cases), and neck/cervical spine CT scan (31.6%). For 74 cases of old WAD, 10 imaging tests were ordered, most commonly neck/cervical spine X-ray (*n* = 4).

#### Prescribed medication for patients diagnosed with Whiplash Associated Disorders

One or more medications were recorded for 53.3% of WAD problems, which is 75.2 per 100 WAD problems (*n* = 103 medications) (62.2 per 100 old and 90.5 per 100 new WAD cases). Of these, 71.8% were prescribed, 1.9% were supplied by the GP, and for 26.2%, the GP advised over-the-counter (OTC) purchase. 49.2 medications were prescribed per 100 new cases (95% CI: 31.9–66.5), and 58.1 per 100 old cases (95% CI: 28.0–88.3). Over the counter (OTC) medications were predominately recommended for new cases (92.6% of OTC medications).

Considering all WAD cases together, the most common types of prescribed medications were analgesics (*n* = 42, 30.7 per 100 WAD problems) and anti-inflammatory and anti-rheumatic drugs (*n* = 16, 11.7 per 100 WAD problems). Of the analgesics, 16 (11.6 per 100 WAD problems) were opioids (oxycodone, tramadol, fentanyl, buprenorphine), and 28 (20.4 per 100 WAD problems) were other analgesics and antipyretics (paracetamol, paracetamol/codeine, paracetamol/caffeine, paracetamol/buprenorphine). The anti-inflammatory drugs were ibuprofen, diclofenac, naproxen, celecoxib, and meloxicam. Other prescribed medications were anxiolytics (*n* = 9, 6.6 per 100 WAD problems), systemic corticosteroids (*n* = 2, 1.5), muscle relaxants (*n* = 2, 1.5), antiepileptics (*n* = 2, 1.5) and antidepressants (*n* = 1, 0.7) (Table 3).

**Table 3:** Prescribed medications classified by ATC Level 3

ATC label	n	Per 100 WAD problems (95% CI)
Analgesics	42	30.7 (17.4–43.9)
Anti-inflammatory and anti-rheumatic products	16	11.7 (6.1–17.2)
Anxiolytics	9	6.6 (1.9–11.3)
Systemic corticosteroids	2	1.5^a^
Muscle relaxants	2	1.5^a^
Antiepileptics	2	1.5^a^
Psychoanaleptics	1	0.7^a^
Total	74	54 (36–72)

Over-the-counter and GP supplied medications have not been included

^a^95% CIs not calculable due to low frequency

Non-recommended drugs [10] accounted for 21.7% of medications prescribed for WAD problems, at a rate of 11.8 per 100 WAD problems. The most common was diazepam (6.6 per 100 WAD problems); those infrequently prescribed were corticosteroids, muscle relaxants and anticonvulsants (1.5 each) and anti-depressants (0.7).

Table 4 displays the types of medications GPs prescribed for WAD. The most common classes of drugs were

- simple analgesics: 19.7 per 100 WAD problems (paracetamol 8 per 100 WAD problems; NSAIDS 11.7 per WAD problems),
- compound analgesics: 12.4 per 100 WAD problems (opioid containing 10.2 per 100 WAD problems; nonopioid containing 2.2 per 100 WAD problems), and
- opioids: (11.6 per 100 WAD problems).

**Table 4:** Medications prescribed for Whiplash Associated Disorders by generic name

Classification	Generic		n	Per 100 WAD problems (95% CI)
*Simple analgesics*				
Paracetamol	Paracetamol	11		8 (3.2–12.9)
NSAID	Ibuprofen		5	3.6 (0.5–6.8)
	Diclofenac sodium		4	2.9 (0.1–5.8)
	Naproxen		3	2.2 (0.0–4.6)
	Celecoxib		2	1.5^a^
	Meloxicam		2	1.5^a^
	Total	16		
*Compound analgesic (opioid)*	Paracetamol/Codeine	14		10.2 (5.2–15.3)
*Compound analgesic* *(nonopioid)*	Paracetamol/Caffeine		1	0.7^a^
	Orphenadrine/Paracetamol		2	1.5^a^
	Total	3		
*Opioid*	Oxycodone		8	5.8 (0.3–11.4)
	Fentanyl		2	1.5^a^
	Tramadol		5	3.6 (0.0–7.4)
	Buprenorphine		1	0.7^a^
	Total	16		
*Benzodiazepine*	Diazepam		9	6.6 (1.9–11.3)
*Steroid*	Prednisolone		2	1.5^a^
*Anti-convulsant*	Pregabalin		1	0.7^a^
	Topiramate		1	0.7^a^
	Total	2		1.4
*Antidepressant*	Amitriptyline		1	0.7^a^
	Total	74		54 (36–72)

Over-the-counter and GP supplied medications have not been included

^a^95% CIs not calculable due to low frequency

The most commonly prescribed individual medications were paracetamol/codeine (10.2 per 100 WAD problems), paracetamol (8), diazepam (6.6) and oxycodone (5.8). Opioids/opioid containing compound analgesics were prescribed at a rate of 21.8 per 100 WAD problems.

#### Other non-pharmacological treatments

Clinical treatments for WAD overall included counselling (6.6 per 100 WAD problems), general advice/education, (5.8), sickness certificate (5.1), and other administrative document (4.4). GP-provided procedural treatments included physical medicine/rehabilitation (16.1 per 100 WAD problems), other therapeutic procedures (2.2) and local injection (1.5).

#### Referrals

More than 80% of referrals (82.9%, *n* = 29) were to physiotherapists (21.2 per 100 WAD problems). Only 2 patients were referred to medical specialists– one orthopaedic surgeon and one neurosurgeon.

## Discussion

WAD lead to substantial personal and economic burden worldwide. As patients with WAD are commonly managed in primary care, it is important to determine the nature of care delivered and whether it is consistent with current recommended practice. Ours is the first study to explore Australian GP management of WAD using a nationally representative sample. We found that from 2013 to 2016, GPs saw patients with WAD problems at a rate of 5 per 1000 GP-patient encounters for females and 4 per 1000 for males. In a previous study [18] using BEACH data from 2000 to 2010, Australian GPs managed new neck pain problems at a slightly lower rate of 3 per 1000 of GP-patient encounters. It is difficult to compare the two studies directly because new neck pain included other causes besides WAD (for example non-traumatic neck pain), and our study included both old and new cases of WAD, with new defined as first presentation and old as subsequent presentations.

### Imaging

Current clinical guidelines for the management of acute WAD recommend the use of the Canadian C-Spine rule to decide whether cervical spine X-ray is needed to rule out WAD IV – a fracture or dislocation [10]. This rule is very specific and sensitive for detecting WAD IV [19]. For WAD Grades I and II imaging is not necessary; imaging is recommended for WAD Grade III if there is suspected neurological damage [10].

The GPs in our study ordered imaging for 21.2% of patients with WAD, a similar rate to that reported by Michaleff et al. where imaging was provided for 22.8% of patients with general neck pain. In both studies the vast majority were for plain x-rays with ultrasound and computerised tomography requests being uncommon [18]. However, the rates found in both studies were lower than those found in the analysis of a compensation claim database where 34% of patients lodging a WAD claim in Victoria, Australia were referred for imaging [20]. The findings of the Australian studies suggest greater rates of imaging for WAD and neck pain by Australian GPs compared to data from other countries. Vos et al. reported that 9% of people with neck pain were referred for imaging in The Netherlands, however this study was conducted 10 years ago and it is possible that imaging rates may have increased there over this time [21].

The imaging rates seen in Australia for neck pain following MVC’s are a concern. The excessive use of imaging exposes patients to unnecessary radiation, increases health costs and may even prolong the duration of disability [22], albeit this data is from low back pain. Reasons for increased referral for imaging by GPs may be related to concerns about litigation, patient demand or lack of confidence in diagnosing neck pain [18]. A recent cross-sectional survey of 423 Australian GPs revealed that GPs generally were not aware of indications for cervical spine Xray after neck injury. Only 44% of respondents correctly identified that inability to rotate the neck beyond 45° to the left or right was an indicator for cervical spine x-ray as per the Canadian C-Spine Rule algorithm [12].

### Clinical/procedural treatments

Clinical treatments included advice, education, counselling, and reassurance. Procedures included all physical treatments (i.e. manual therapy, injection and splinting). Up to two procedures, other treatments or counselling were recorded per encounter, only including those actually provided at the encounter.

Clinical/procedural treatments for WAD provided by GPs in our study included GP-provided physical medicine/rehabilitation (16.1 per 100 WAD problems), counselling (6.6 per 100 WAD problems), general advice/education (5.8), other therapeutic procedures (2.2) and local injection (1.5).

Current clinical guidelines [10] recommend that the most important aspect of management of acute WAD is providing assurance and encouragement to return to normal activities and exercise [23], but GP provided advice/education was only documented in 5.8 per 100 WAD problems in our study. It is not yet clear whether assurance and advice alone will improve long-term outcomes or prevent the development of chronic pain. Local injection, which is not recommended, was used in 1.5 per 100 WAD problems. Further education of GPs about the levels of evidence for various management options for WAD is recommended.

The cross-sectional survey of Australian GPs revealed a knowledge gap regarding appropriate treatment for WAD: 80% incorrectly thought that manipulation is a first line evidence-based treatment [12]. Rather, first line evidence-based treatments are: Reassure and stay active; return to usual activities; and range of motion, low load isometric, postural endurance and strengthening exercises [10]. The guidelines state: “*Practitioners may provide thoracic manipulation for the treatment of acute WAD. However, thoracic manipulations should only be provided by registered health practitioners trained in the specific methods and in accordance with current professional standards. There is no evidence for the efficacy of cervical manipulation in the treatment of acute WAD*” [10].

Whilst we cannot identify GPs’ use of or preference for manipulation treatment, the rate of GP-provided physical medicine/rehabilitation therapies (16.1 per 100 WAD problems), and the rate of referral for physical therapies (82.9% of 25.5 referrals per 100 WAD problems (*n* = 29) were to physiotherapists (21.2 per 100 WAD problems)), may indicate that manipulation is perceived as being beneficial for WAD. The guidelines state: “*Practitioners may provide manual therapy as it may be effective for the treatment of acute WAD. Manual therapy*, defined as a clinical approach utilizing specific hands-on techniques, including but not limited to manipulation, mobilization, and massage, *can be used in conjunction with exercise and advice, if there is evidence of continued benefit via validated outcome measures*” [10]. Our study showed a similar rate of referral to Michaleff’s study of neck pain [18] in which 20.3% of cases were referred elsewhere (to allied health practitioners including physiotherapists, and specialists) [18]. A higher rate of referral to physiotherapists experienced in the treatment of WAD is recommended, especially for patients at high risk of ongoing severe pain and disability (those 35 years or over, having an initial NDI score of 40% or over, and the presence of hyperarousal symptoms [24]).

### Medication

There is no high level evidence on the effectiveness of medication for the management of WAD, with a recent review finding no RCTs which supported the use of any medication for WAD [11]. Based on consensus, Clinical Practice Guidelines for the management of acute WAD recommend “*regular paracetamol and if this is ineffective, then NSAIDS may be used. Oral opioids, preferably short-acting agents at regular intervals, may be necessary to relieve severe pain in the treatment of acute WAD, but ongoing need for such treatment requires reassessment*” [10].

NSAIDs were the most common medications used by GPs in our study at 11.7 per 100 total WAD problems, followed by paracetamol alone at 8 per 100 WAD problems. This is consistent with a previous Australian study of neck pain (not specifically WAD) that found the medication most often recommended for the treatment of new neck pain was NSAIDs followed by paracetamol [18]. However, that Australian GPs seem to prescribe NSAIDS over paracetamol is a concern considering the well-known link with gastrointestinal side effects.

While the clinical guidelines call for cautious use of opioids [10], compound analgesics containing paracetamol and opioids were used at a rate of 10.2 per 100 WAD problems, and opioids alone comprised 16/74 medications prescribed (11.6 per 100 WAD problems). Opioids alone were prescribed in 21.7 per 100 old WAD cases. It is not possible to separate acute from chronic WAD in this study, because “new” means “first presentation” in this context. The use of opioids in this patient group is a concern. After an initial period of analgesia, opioids are known to cause hyperalgesia [25]. Consistent with these data, genetic epidemiologic studies suggest that endogenous opioids at the time of traumatic stress such as motor vehicle crash worsen pain outcomes [25]. In addition, early prescription of opioids may increase the risk of chronic opioid use, which is associated with opioid dependence and abuse. The misuse of prescription opioids in the US and Canada has become a public health crisis, with evidence that a similar problem is developing in Australia [26]. US data also show that early provision of opioids in the hospital emergency department for motor vehicle crash injury patients is associated with continued use 6 weeks later [25].

We found that the rate of opioid prescription by Australian GPs was similar to the rate of referral to physiotherapy. Physiotherapy in the form of exercise and activity has the strongest evidence base for the treatment of WAD [10] and is a much safer treatment option than opioid medication. Our findings indicate a need for further education of GPs and the general public, in relation to more informed use of opioids for WAD.

The medication management guidelines are as follows:

#### Clinical guidelines for the management of WAD

*“For WAD grade I, no medication other than simple analgesics should be prescribed. For WAD grades II and III, non-opioid analgesics and NSAIDs can be used to alleviate pain in the short term. Their use should be limited to three weeks and should be weighed up against known side-effects, which appear to be dose related. Opioid analgesics are not recommended for patients with WAD grade I. They may be prescribed for pain relief in patients with acute WAD grades II and III experiencing severe pain (VAS >8) for a limited period of time. Psychopharmacologic drugs are not recommended in patients with acute and subacute WAD of any grade. However, they can be used occasionally”* [10]*.*

Drugs *not* recommended by the guidelines were used at a rate of 11.8 per 100 WAD problems (comprising 21.7% of medications prescribed for WAD problems), the most common being diazepam (6.6 per 100 WAD problems) with corticosteroids, muscle relaxants and anticonvulsants infrequently used (1.5 per 100 WAD problems each), as were anti-depressants (0.7 per 100 WAD problems). Although the guidelines state that practitioners should not prescribe muscle relaxants, anti-convulsants and anti-depressants because they are not effective in the management of acute WAD [10], the GPs in this study prescribed diazepam at a rate of 6.6 per 100 WAD problems. Though no studies of benzodiazepines for neck pain were found, in a recent trial in ED patients with acute, non-traumatic, non-radicular low back pain, the combination of naproxen and diazepam did not improve functional outcomes or pain compared with the combination of naproxen and placebo at 1 week and 3 months after discharge from ED [27]. Overprescribing of benzodiazepines and the various harms resulting from this is of concern. In the 20-year period from 1992 to 2011, 174,080,904 benzodiazepine scripts were written in Australia. Temazepam (35% of scripts) and diazepam (23%) were the most frequently dispensed benzodiazepines [28]. Long-acting benzodiazepine consumption, especially among older people, has implications for mortality, morbidity and cost-effective prescribing [29].

The higher than recommended use of certain medications may result from prior use before the motor vehicle crash injury. A recent study found that 12% of participants were already taking antidepressants prior to their injury, so less than half of antidepressant use post-injury resulted from the incident injury [30]. Similarly, prescription opioid and benzodiazepine use before motor vehicle crash (MVC) injury was substantial [31]. This may be due to increased MVC risk because of being on these medications [32, 33]. Therefore the significance of post-injury prescription drug use cannot be established without taking pre-injury use into account. Unfortunately as our study was cross-sectional we do not have this information and cannot exclude that patients may have been taking these medications prior to their injury.

### Strengths and limitations of the study

Strengths of this study include the stringent data management procedures used and the nationally representative sample. Thus our findings provide an accurate picture of the way GPs manage WAD in Australia.

There are several limitations. Patients whose WAD is undiagnosed would be missed by our methods. No data were collected on time since injury, so our figures include both new and old whiplash injuries. This is relevant because treatment is often different once the condition becomes persistent or chronic. No data were collected on whether the medication usage existed prior to the injury.

In the BEACH study, procedural and clinical treatments were recorded by using free text. This may have resulted in under estimation of the use of these treatments. Similarly, some GPs recognised advice-giving as a specific aspect of management and recorded it separately, while others considered giving advice to be part of usual care and may not have recorded this separately.

## Conclusion

WAD result in a large personal and economic burden, so the impact of improvements in GP management is potentially significant. GPs referred about 21% of new cases for imaging, and prescribed a range of drugs, approximately 22% of which were outside clinical guidelines. Advice/education was a recommended but underused treatment for WAD. Local injections, muscle relaxants, anti-convulsants, benzodiazepines and anti-depressants, for which there is no evidence for efficacy and which are not recommended, were overused. Though opioid analgesics are not recommended treatment for patients with WAD grade I but can be used for a limited period of time in patients with acute WAD grades II and III and severe pain (VAS >8) [10], in this study opioids alone were prescribed in 21.7 per 100 of second or subsequent presentation WAD cases. Non-recommended treatments were used in a total of 34.4 per 100 WAD cases. This data suggests need for further education of GPs, including indications for imaging after whiplash injury (Canadian C-spine rule); identification of those at high risk of incomplete recovery); evidence based therapies for WAD and clinical management guidelines. In particular, further education needs to include the value of advice/education, the need to prescribe simple analgesics rather than ineffective medications such as local injections, muscle relaxants, anti-convulsants, benzodiazepines and anti-depressants, and the cautious use of opioids, for this condition.

## Abbreviations

ATC: Anatomical Therapeutic Chemical
BEACH: Bettering the Evaluation and Care of Health
CI: Confidence Interval
ED: Emergency Department
GPs: General Practitioners
ICPC-2: International Classification of Primary Care-2
MVCs: Motor Vehicle Crashes
NSAIDs: Nonsteroidal anti-inflammatory drugs
OTC: Over the counter
TAC: Transport Accident Commission
WAD: Whiplash Associated Disorders
